# Development of liver disease caused by chronic periodontitis in rats

**DOI:** 10.1590/1678-7757-2025-0550

**Published:** 2025-11-17

**Authors:** Karen Neisman Rodriguez AYALA, Vinícius da Silva CAETANO, Any Carolina Cardoso Guimarães VASCONCELOS, Maria Vitoria Pereira de SOUSA, Nikaely Brandão BARBOSA, Luanna Maria Soares MESQUITA, Paulo Roberto Carneiro GOMES, André Luis dos Reis BARBOSA, Hélio Mateus Silva NASCIMENTO, Daniel Fernando Pereira VASCONCELOS

**Affiliations:** 1 Universidade Federal do Piauí Programa de Pós-Graduação em Odontologia Teresina PI Brasil Universidade Federal do Piauí, Programa de Pós-Graduação em Odontologia, Teresina, PI, Brasil.; 2 Universidade Federal do Delta do Parnaíba Laboratório de Análise e Processamento Histológico Parnaiba PI Brasil Universidade Federal do Delta do Parnaíba (UFDPar), Laboratório de Análise e Processamento Histológico (LAPHis), Parnaiba, PI, Brasil.; 3 Instituto de Educação do Vale do Parnaíba Faculdade de Medicina Parnaiba PI Brasil Instituto de Educação do Vale do Parnaíba (Afya-Parnaíba), Faculdade de Medicina, Parnaiba-PI, Brasil.; 4 Universidade Federal do Delta do Parnaíba Laboratório de Fisiologia-Farmacologia Experimental Parnaíba PI Brasil Universidade Federal do Delta do Parnaíba (UFDPar), Laboratório de Fisiologia-Farmacologia Experimental (LAFFEX), Parnaíba, PI, Brasil.

**Keywords:** Fatty liver, Histopathology, Periodontal disease, Oxidative stress, Steatosis

## Abstract

Periodontitis is an inflammatory disease characterized by producing alterations in periodontal tissues, but it has been associated with the manifestation of systemic alterations, related to those produced in liver tissue. This study aims to evaluate the progression of liver damage caused by ligation-induced periodontitis in rats with time intervals of ten to 80 days.

**Methodology:**

Overall, 40 rats were divided into five groups: control (no ligatures), P10, P20, P40, and P80 (teeth with ligatures at intervals of ten, 20, 40, and 80 days) in which we verified liver disease caused by periodontitis. Oral parameters were evaluated: gingival bleeding index (GBI), probing pocket depth (PPD), dental mobility (TM), myeloperoxidase activity (MPO), alveolar bone height (ABH). Liver parameters were evaluated: liver weight, histopathological scores for steatosis, inflammation, and necrosis in the liver; glutathione (GSH) and malondialdehyde (MDA). Serum parameters were also evaluated: concentrations in liver tissues, blood levels of albumin, aspartate aminotransferase (AST), alanine aminotransferase (ALT), glucose, total cholesterol, and total proteins.

**Results:**

The results showed that the hepatic steatosis score gradually increased (P<0.05) in rats with induced periodontitis for up to 20 days (P20); and the rats with ligatures for 40 (P40) and 80 days (P80) had stable scores compared to the P20, without any further worsening, similarly occurred with GSH, MDA, and total cholesterol.

**Conclusions:**

This study indicated that liver alterations caused by ligature-induced periodontitis are progressive in early stages (0-20 days) and reach a plateau in later stages (40-80 days).

## Graphical abstract

**Figure:**
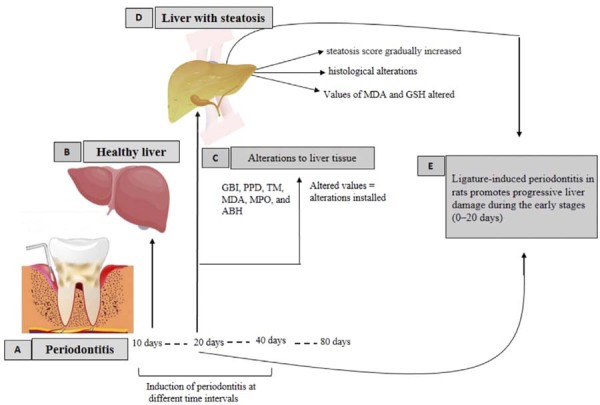

## Introduction

Periodontitis is an inflammatory disease, characterized by a progressive destruction of the periodontal tissue around the dental root, influenced by several host, immune, and environmental factors,^1,2^ affecting thousands of people in Latin America,^3^ the United States of America,^4^ and some European countries such as Germany.^5^

Periodontitis not only causes changes in periodontal sites, but it can also promote damage to systemic organs, either via direct mechanisms (blood circulation, lymphatics) or via indirect mechanisms (such as the release of inflammatory molecules from the host and ROS agents reactive to oxygen, currently being associated with multimobility.^6,7^

Furthermore, several studies report the association of periodontitis with systemic diseases such as diabetes,^8^ obesity,^9^ atherosclerotic cardiovascular disease (Herrera et al.,^7^ 2023), and liver disorders. Evidence points to an association to elevated immunological activity, due to the release of inflammatory mediators from monocytes, subsequently, release of pro-inflammatory mediators, that can affect the susceptibility and progression of other systemic diseases.^10-13^

According to the associations, studies focusing on the relationship between periodontal and systemic conditions have shown that hepatic steatosis has an important association, evidencing a bidirectional relationship between the two diseases. In parallel to this, other studies have also demonstrated that lipopolysaccharides (LPS) from periodontal pathogens function as periodontitis inducers in different cells^14^ and can promote liver disorders.^15^ Nevertheless, our research team has shown that ligatures placed on the lower molars could induce periodontitis and were sufficient to cause liver disorders,^12,16^ as observed in hepatic steatosis and its association with oxidative stress that can be reversed by the elimination of local irritants (in this case, the ligatures).^13^

However, the time required for periodontal damage and systemic repercussions resulting from ligatures is not yet well defined. Therefore, our hypothesis is to clarify how long it takes for damage to occur and whether a plateau is reached during the intervention, measured in days.

In view of the high prevalence of periodontitis^3,4,5^ and the lack of information on the progression of liver alterations during periodontitis, this study aimed to evaluate the progression of liver alterations in rats with ligature-induced periodontitis.

## Methodology

### Animals

A total of 40 adult female Wistar rats *(Rattus norvegicus*), weighing 250±50.2 g, were used for experimental procedures. The rats were kept in standard condition with a light-dark cycle of 12 hours at 23±2°C, relative humidity of 55±10%, with free access to water and food (ad libitum). All treatments and procedures with animals were performed in accordance with the Guide for the Care and Use of Laboratory Animals (National Institute of Health, Bethesda, MD, USA) and the guidelines of the Institutional Committee of Animal Ethics (protocol number 384/17).

### Sample size calculation

A sample size calculation was performed a priori to ensure adequate statistical power for detecting biologically relevant differences among the groups. Sample size estimation was conducted using G*Power 3.1 for a one-way ANOVA design (fixed effects, omnibus test). The analysis assumed a two-sided alpha level of 0.05, a statistical power (1−β) of 0.95, and an effect size *f*=0.6 (corresponding to a large effect according to Cohen’s conventions), based on previous studies in ligature-induced periodontitis models^11,12^ and pilot data using identical procedures and outcome measures. Under these parameters, the calculation indicated that n=8 animals per group would provide sufficient power (95%) to detect the expected differences.

### Experimental design

The rats were randomly divided into five groups of eight: Control (no ligation), P10 (teeth linked for ten days), P20 (teeth linked for 20 days), P40 (teeth linked for 40 days) and P80 (teeth linked for 80 days). Periodontitis was induced after general intramuscular anesthesia with 15 mg / kg of 2% xylazine hydrochloride (Francotar-Virbac^®^, Roseira, SP, Brazil) and 35 mg / kg of ketamine (Rompum-Bayer^®^, SP, Brazil). A nylon ligature (3-0) (Shalon^®^, Goiania, GO, Brazil) was placed around the cervical region of the first right and left lower molar of each rat and then firmly knotted.^11,12^

Blood samples were collected from the retro orbital plexus for biochemical tests after the time intervals of induction of periodontitis, then the rats were euthanized, and the total body and liver weights were calculated. To determine the degree of chronicity of periodontitis lesions and their impact on the liver, histochemical, blood, oral, and hepatic parameters were evaluated.

### Oral parameters: GBI, PPD, MPO, ABH, and TM

Gingival bleeding index (GBI): the periodontal pockets or the gingival groove of the first lower molars were probed for ten seconds and classified according to Liu, et al.^17^(2012), in scores ranging from 0 to 5 in which score 0: the margin Gingival and gingival papilla (GMP) is healthy and no bleeding is observed after mild probing. Score 1: the gingival margin and the gingival papilla are slightly inflamed, and no bleeding is observed after probing. Score 2: the gingival margin and the gingival papilla are slightly inflamed; changes in color, absence of edema, and dotted hemorrhage occur after a slight probing. Score 3: the gingival margin and the gingival papilla are moderately inflamed; alterations in color, mild edema, and bleeding after a slight catheter while the blood is still in the gingival crack are visible. Score 4: the gingival margin and the gingival papilla are severely inflamed; changes in color, severe edema, and bleeding after probing while blood flow is observed outside the gingival groove. Score 5: the gingival margin and the gingival papilla are severely inflamed; There are changes in color, severe edema, ulcer, bleeding after probing, spontaneous bleeding outside the gingival groove.^18,19^

For the evaluation of PPD, a round tip probe (0.2 mm tip radius) was used, three points (mesiovestibular, distovestibular, and medialbucal) were evaluated, and measurement was performed.^18,19^

MPO activity was evaluated by the accumulation of neutrophils in the gingival tissue (soft tissue) around the lower first molar. In summary, 40 mg of gingival tissue at 50 mg / ml were homogenized in potassium buffer containing 0.5% hexadecyl trimethyl ammonium bromide (Dinamica^®^, Diadema, SP, Brazil). The mixture was centrifuged at 39,000 g for five minutes at 4°C. The sediment was resuspended and MPO activity was evaluated by measuring the variation in absorbance at 450 nm using o-dianisidine dihydrochloride and 1% hydrogen peroxide. MPO activity was described as units / mg of tissue. One unit of MPO activity was defined by the conversion of 1μmol of hydrogen peroxide into water in 60 seconds at 24 ° C, as described by Chaves.^19^

### Alveolar bone height (ABH) evaluation

Measurement of alveolar bone height (ABH) was evaluated as follows: to delimit the cement-enamel junction (CEJ), the jaws were stained with methylene blue (1% aqueous) after dissecting the soft tissue. The image of the alveolar bone height for each hemimandible was captured using a stereoscopic microscope with a magnification of 30x. Three points were measured in the lingual part for the evaluation of the mean height of the alveolar bone, which were made along the root axis, as follows: a) ABH-1, the distance was obtained by measuring the height from the cement-enamel junction to the alveolar ridge, in the anterior (mesial) portion of the root of the lower first molar; b) ABH-2, the distance was obtained by measuring the height from the cement-enamel junction to the alveolar ridge, at the middle root of the lower first molar; c) ABH-3, the distance was obtained by measuring the height from the cement-enamel junction to the alveolar ridge, at the distal root of the lower first molar. Finally, the following formula was used to calculate the sum of three ABH:

ABH = ABH-1/3 + ABH-2/3 + ABH-3/3

These images were quantified using image analysis software ImageJ v.1.48 (NIC, USA).^12^

### Evaluation of tooth mobility (TM)

Mobility of the first lower (TM) or molars was scored as follows: 0=lack of mobility, 1=mild mobility (vestibular-palatal), 2=moderate mobility (vestibular-palatal and mesial-distal) and 3=severe mobility (the tooth moves in and out of the socket)^20^.

### Histopathological evaluation of the liver

Samples were extracted, cut into fragments and fixed in 10% buffered formaldehyde. After being dehydrated in increasing concentrations of ethyl alcohol and immersed in xylol, the fragments were immersed in paraffin. The 5µm thick sections were stained with hematoxylin and eosin (HE), before evaluation with optical microscopy (Zeiss, Oberkochen, Germany).

Histological evaluation of liver tissue and inflammation were scored as follows: 1) steatosis (the percentage of liver cells with fat): score 0: absent or present in <5% of hepatocytes; score 1:>5% and <25%; score 2:>25% and <50%; score 3:>50% and <75%; and score 4:>75%. 2) Inflammation and necrosis: 0: none; 1:<2 spotlights / field; 2: 2-4 spotlights / field; 3:>4 spotlights / field.^21^

### GSH and MDA concentration in liver

The concentration of GSH for liver tissues was determined according to the method described by Sedlak and Lindsay.^22^ MDA levels for liver and gingival tissues were performed to assess lipid peroxidation according to Mihara and Uchiyama^23^ (1978), briefly, tissue homogenates were reacted with phosphoric acid and thiobarbituric acid (TBA), heated in boiling water, and extracted with n-butanol. The absorbance of the butanol phase was measured at 535 and 520 nm, and the difference between these readings was taken as the TBA value.

### Serum albumin, ALT, AST, glucose, total cholesterol, and total protein determinations

Serum content of albumin, ALT, AST, glucose, total cholesterol, and total protein were measured with a marketable ELISA Kit (Labtest, Belo Horizonte, MG, Brazil).

### Statistical analysis

Data were plotted and analyzed using GraphPad Prisma version 7.0^®^ (MA, USA). The results are expressed as mean ± SEM and/or median. Shapiro-Wilk test was used to verify the data distribution. Differences between the three groups were analyzed using ANOVA and the Student-Newman-Keuls test, for parametric data. Also, the Kruskal-Wallis nonparametric test was used, followed by Dunn’s test for multiple comparisons, for non-parametric data. Differences were significant when P<0.05.

## Results

### Oral parameters: GBI, PPD, TM, MDA, MPO, and ABH

**Figure 1:**
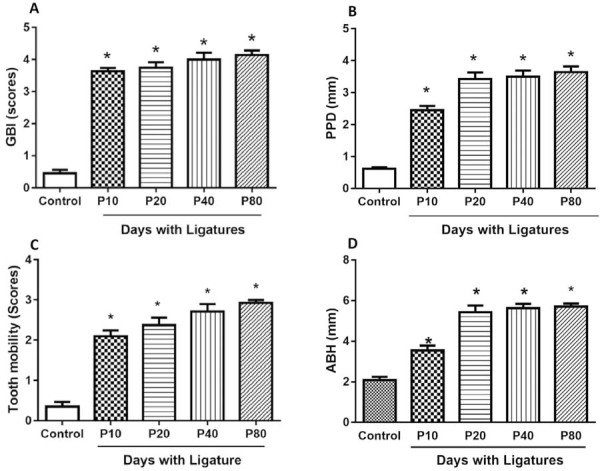
The bar graphs illustrate the mean and standard deviation of the gingival bleeding index (GBI) A; pocket probing depth (PPD) B; dental mobility (TM) C, and alveolar bone height (ABH) D; in which there are significant differences of the periodontitis groups with respect to the control group.

The results on GBI, PPD, TM, MDA, MPO, and ABH showed a significant increase in the measurement of these parameters by comparing the control group with the P10, P20, P40, and P80 groups (Figure 1A-D, respectively).

First, GBI values showed significant differences between the control group and the periodontitis groups (Control=0.45±0.51, P10=3.63±0.49, P20=3.75±0.46, P40=4.00±0.58, P80=4.14±0,38; P<0.05), thus showing the beginning of the gingival alterations, obtaining a score of scale 3 for group 10, starting with marginal-papillary inflammation, edema, and bleeding at probing, and from group 20 increase more these own patterns of a punctuation 4 in which there is already beginning of exponential bleeding, while this scale is maintained in groups 40 and 80.

Values for PPD (Control=0.61 mm±0.21, P10=2.45±0.60, P20=3.43±0.53, P40=3.50 mm±0.5, P80=3.64±0.47; P<0.05). The dental mobility score (Control=0.34±0.57, P10=2.1±0.64, P20=2.37±0.52, P40=2.71±0.48, P80=2.92±0.26; P<0.05), shows the manifest difference of control groups with respect to the periodontitis group, reaching moderate to severe mobility scores.

Significant differences were shown in the ABH values of the periodontitis groups in relation to the control group (Control=2.09±0.38, P10=3.55±0.58, P20=5.42±0.81, P40=5.62±0.64, P80=5.70±0.37) this difference remained until the 20-day group.

To measure the neutrophil infiltrate, the neutrophilic activity test was performed and showed high levels of MPO in gingival tissue of groups P20 and P40 (Control = 3.49 UMPO / mg of tissue ±3.44, P20=51.68 UMPO / mg of tissue ±19.47, P40=56.32 UMPO / mg of tissue ± 10.48, P<0.05 respectively) with significant statistical difference.

### Histopathological evaluation of the hepatic tissue

**Figure 2:**
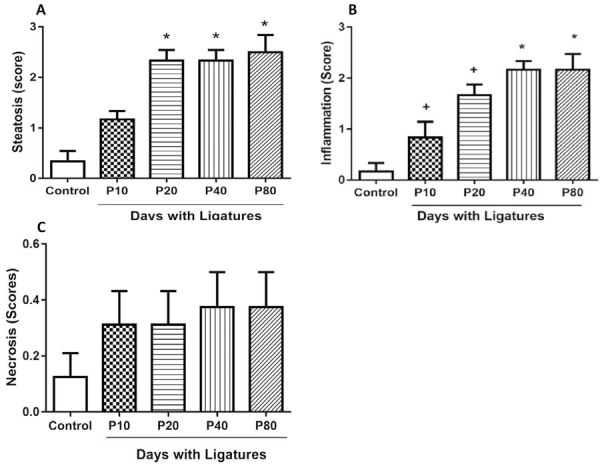
The bar graphs illustrate the mean and standard deviation of the histopathological evaluation indexes of the liver in which there is a marked difference of the periodontitis groups with the control group A; for the values of inflammation, an increase in this manifestation is seen from the P20 group, stabilizing on days 40 and 80 B; not being shown in the necrosis indices, in which there are no significant differences, showing two or no necrotic focus C.

**Figure 3:**
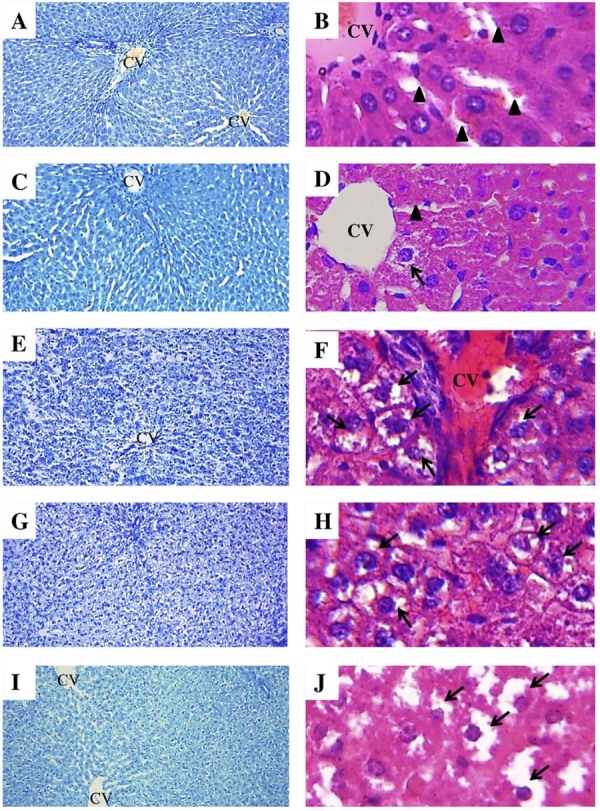
Histopathological evaluation of the hepatic tissue. A) and B) represent the hepatic tissue of the control group without histologic changes. C) and D) demonstrate the liver of the P10 group that hepatocytes showed abnormal structure with loss partly of organization cordons around the central vein (CV). E) and F) illustrate the liver of the P20 group that hepatocytes showed abnormal structure with loss of organization cordons around the central vein (CV) and the arrows indicate hepatocytes with steatosis, like the P40 group in G) and H). The groups 80 in I) and J) demonstrate steatosis pointed by the arrow into hepatocytes, similarly to the other group with periodontitis. Toluidine blue stain in A, C, E, G, and I, hematoxylin and eosin in B, D, F, H, and J; CV, central vein; arrowheads indicate blood vessel; original magnification 60x of images A, C, E, G, and I; 600x of images B, D, F, H, and J.

The control group had the lowest significant steatosis score with respect to the periodontitis groups P20, P40, and P80 (Control=0.33±0.51, P10=1.17±0.41, P20=2.33±0.52, P40=2.33±0.52, P80=2.5±0.84) (Figure 2A); histologically, the disorganization of hepatocytes can be observed from the ten-day group, not showing distinction on days 40 and 80 (Figures 3A-J). Inflammation score values (Control=0.17±0.41, P10=0.83±0.75, P20=1.66±0.51, P40=2.16±0.51, P80=2.17±0.75) show that the differences between the groups only manifested from 20 days of induction, showing its greatest exposure in groups 40 and 80 days compared to the control group, but not among them, which translates into the existence of two to more than four foci of liver inflammation (Figure 2B). As for necrosis (Control=0 (0-1), P10=0 (0-1), P20=0 (0-1), P40=0 (0-1), P80=0 (0-1) P<0.05) the values did not differentiate with respect to the control group and between the periodontitis groups (Figure 2C).

The total weight of the animal showed significant differences increasing this from the periodontitis group 20 days with respect to the control group and from this one with respect to the 40- and 80- days groups (Table 1).

**Table 1:** Weight parameters evaluated.

	Days of induction				
Groups	Control	P10	P20	P40	P80
Body(g)	145.6±11.3	158 ±7.87	167.3±12.1*	186.3±0.11*	204.3±17.1*
Liver Absolute (g)	6.96±0.91	7.19±0.37	7.43±0.3*	7.50±0.53*	7.4 ±0.45*

*P<0.05 indicates comparison to the control group and P<0.05 indicates comparison with periodontitis groups.

### GSH concentration of liver and MDA levels of liver and gum

**Figure 4:**
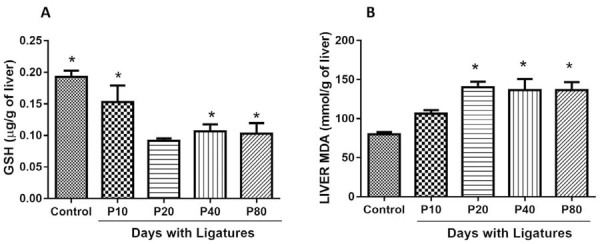
The bar graphs illustrate the mean and standard deviation of the GSH and MDA indices, with a decrease in Glutathione values in liver tissue A, a sign that there is an increase in lipid peroxidation that results in high oxidative stress values that can be noticed according to the MDA indices B.

The GSH concentration of liver tissue samples in P20 was significantly lower than the control group (Control=0.19 μg / g of liver ±0.01, P10=0.15 μg / g of liver ±0.07, P20=0.09 μg / g of liver ±0.01, P40=0.11 μg / g of liver ±0.03, P80=0.10 μg / g of liver ±0.04; P<0.05). Although there was a decrease in the concentration of GSH in the P20 group, the measurement of this oxidative stress marker did not show significant differences with respect to the concentration of GSH in the P40 and P80 groups. (Figure 4A).

Similar results were found in MDA levels, in which the control group showed a significantly lower value compared to groups P20 and P40 with respect to liver tissue, group 80 showed similar scores with group 40 (Control = 79.95 mmol / g of liver ±8.45, P10=106.4 mmol / g of liver ±.55, P20=140.3±21.5 mmol / g of liver, P40 =136.4 mmol / g of liver ±40.66, P80=136.3±25.86; P<0.05). (Figure 4B).

### Serum albumin, ALT, AST, glucose, total cholesterol, and total protein determinations

Data on the determinations of serum albumin, ALT, AST, glucose, total cholesterol, and total proteins observed in (Table 2), in which the ALT values show non-significant differences in increasing order in relation to the days of exposure to periodontitis.

**Table 2:** Blood and liver biomarkers.

		Days of induction			
Groups	Control	P10	P20	P40	P80
Glucose	248.0±6.5	244.8±4.9	259.2±11.1	253.5± 3.5	263.7±9.7
Total Proteins	7.69±2.59	7.57±2.88	6.81±4.23	6.69±2.08	6.66±1.39
Total Cholesterol	66.34±4.45	81.27±15.94	97.31±24.45*	94. 55±12.81*	94.40±11.67*
ALT	33.5±2.0	39.7±6.3	37.1±5.1	38.4±3.4	42.8±5.1
AST	35.8±1.9	58.3±7.3	56.0±11.2	66.3±5.9*	65.2±10.22

*P<0.05 indicates comparison to the control group and P<0.05 indicates comparison to the periodontitis group. Glucose; Total Proteins; Total Cholesterol; ALT, alanine aminotransferase; AST, aspartate aminotransferase.

increased AST values were shown in group P20, showing the highest value in group P40 in relation to P10, P20, and P80 groups respectively. The biochemical determination of total proteins, except the serum albumin, was high in the P40 group in comparison with the control and P20 groups. Total cholesterol values showed significant differences in groups 20 and 40 in relation to the control group, and stability in the score value of group 80 with respect to group 40, and finally the total protein markers did not show significant differences between any of their groups (Table 2).

## Discussion

This is the first study to demonstrate the evolution of liver alterations caused by induced periodontitis in rats, in addition to highlighting the moment at which such hepatic alterations reach a plateau, with liver alterations remaining stable for up to 80 days in rats with ligatures.

Ligation-induced periodontitis located in the mandibular molars would work correctly and that subsequent studies would give new and different results, such as the decrease in the number of related pericytes with the appearance of microvesicular steatosis, observing that the periodontal inflammation center could trigger liver disorders.^18^ Consequently, more studies on the influence of periodontitis in liver disease with microvesicular steatosis mentioned above were important to indicate that they could be reversed with the timely management of the cause, providing joint support with the application of antibacterial agents for therapy of shock^11,13^, reinforcing the work done by Tomofuji,^24^ concluding that by eliminating the cause, liver damage values return to normal.

Later França, et al.^15^ (2017) would study the association of renal alterations with periodontal disease, thus manifesting a reduction in GSH values and an increase in MDA values, without showing alterations in renal function; the changes were structural and histomorphometric, which shows that there is also release of ROS by defense cells at the renal level, which contributes to the degeneration caused by renal lipoperoxidation, increasing oxidative stress and lipid peroxidation with a reduction of GSH values and an increase in the MDA values of the periodontitis group related to the control group.

Pessoa, et al.^12^ (2018) would demonstrate that the results of microvesicular steatosis would not show significant differences in terms of the extent of liver injury in periodontal inflammatory conditions with respect to the number of localization sites. Continuing with the studies, it was evidenced that the high consumption of a high-calorie diet, high in fat, can aggravate the manifestations of hepatic steatosis caused by the induction of periodontitis due to ligatures, with markedly increasing levels of MDA, GSH, steatosis, HDL, total weight, cholesterol, uric acid, and ALT.^16^

Our research is considered the first study that would demonstrate the course of liver disease caused by ligation-induced periodontitis at different time intervals. In our histopathological analysis together with the biochemical tests applied to the proinflammatory and oxidative stress data, our findings that evidenced the progression of liver disorders during experimental periodontitis in rats in different periods were supported.

When evaluating the activity of MPO (granular component of mononuclear lymphocytes considered an antibacterial enzyme), there is an increase in the values of each group that received ligation-induced periodontitis, mainly in groups P20 and P40, infiltrate Neutrophils promotes the destruction of the periodontium with the release of enzymes and reactive oxygen species (ROS).^25^ Periodontal inflammation is not limited to local oral sites and reflects systemic abnormalities due to the release of inflammatory mediators and ROS in the blood.^26^

The levels of MDA in gingival tissue showed that inflammatory periodontal disease promotes lipid peroxidation, these findings were observed in the sample of groups that received ligation-induced periodontitis; MDA is an important marker for oxidative stress and is described with high levels during periodontitis.^27^ MDA levels were high in the P10 group, reaching values peaks in groups P20 and P40, and remained stable in group 80 in relation to group 40 (Figure 4B), showing significant differences in relation to the control group.

Our results show a progression of liver tissue damage, which shows that liver damage by experimental periodontitis in rats could be considered conditioned, showing statistically significant differences in the P20, P40 groups with respect to the control group. Similar findings were identified in the liver concentration in GSH (Figure 4B), GSH (glutathione sulfhydryl) according to the MDA results. The GSH results showed significant differences for the low P20 values in this group, in relation to the control group, in which it was also verified that there were no significant differences between the P40 and P80 groups, in which the values are almost stable between them. (Figure 3A).

The authors suggested that steatosis may evolve to steatohepatitis and fibrosis, which is corroborated by another study in which patients with isolated steatosis had the same progression.^28,29^ In this study, our results did not show significant differences between the P20 and P40 groups in transaminase levels, but these levels were high compared to the control group that began to differentiate from the P10 group and remained high in the 80 group; Although still mild, changes in transaminase levels may indicate underlying conditions that may worsen in the liver,^29^ with steatosis being the initial stage observed in periods of periodontitis^15^ where alterations in serum aminotransferase levels and in liver tissue mosphology^18^ have already been manifested.^26^

Note that the biochemical evaluation showed significant changes in the level of serum albumin that increased in the P20 group (P<0.05), and sometimes serum albumin levels are often not present until the liver injury worsens.^30^ The total cholesterol level also showed an increase in the P10 group, staying high in the 20, 40 groups, observing a decrease in the group 20 of assimilation of the group P80 (P<0.05) (Table 2). Regarding this, some studies were positively correlated with high serum levels during periodontitis.^31^

Nevertheless, it is important to mention the dysbiotic factor, in which it is denoted that Porphyromonas gingivalis-induced periodontitis could induce the progression of non-alcoholic steatohepatitis in rats,^14^ as well as the chronic administration of its endotoxins, such as lipopolysaccharides and protease,^32^ which is a topic that could be further deepened.^33,34^

Our findings represent a pioneering contribution to understanding systemic damage caused by periodontitis, particularly in the liver, using this experimental design. While the study highlights important associations, other mechanisms underlying these alterations may be further clarified via diverse investigative approaches. Additionally, different study designs could provide deeper insights into how hepatic changes develop not only in ligature-induced periodontitis in rats but also in future studies involving humans.

## Conclusion

In conclusion, ligature-induced periodontitis in rats promotes progressive liver damage during the early stages (0–20 days), especially regarding steatosis, inflammation, and biomarkers such as GSH and MDA, with theses alterations reaching a plateau in later stages (40–80 days).
